# Antiretroviral therapy response among HIV-2 infected patients: a systematic review

**DOI:** 10.1186/1471-2334-14-461

**Published:** 2014-08-26

**Authors:** Didier K Ekouevi, Boris K Tchounga, Patrick A Coffie, Joseph Tegbe, Alexandra M Anderson, Geoffrey S Gottlieb, Marco Vitoria, François Dabis, Serge P Eholie

**Affiliations:** Programme PACCI, site ANRS, Abidjan, Côte d’Ivoire; ISPED, Centre INSERM U897-Epidémiologie-Biostatistique, Université Bordeaux, F-33000 Bordeaux, France; Centre INSERM U897- Epidémiologie-Biostatistique, INSERM, ISPED, F-33000 Bordeaux, France; Département de Santé Publique, Faculté des Sciences de la Santé, Université de Lomé, Lomé, Togo; Département de Dermatologie et des Maladies Infectieuses, Université Félix Houphouët Boigny, Abidjan, Côte d’Ivoire; Services des Maladies infectieuses et Tropicales, CHU de Treichville, Abidjan, Côte d’Ivoire; Departments of Medicine & Global Health, University of Washington, Seattle, USA; HIV Department, WHO, Geneva, Switzerland

**Keywords:** HIV-2, Clinical response, Immunological response, Virological response, Antiretroviral treatment

## Abstract

**Background:**

Few data are available on antiretroviral therapy (ART) response among HIV-2 infected patients. We conducted a systematic review on treatment outcomes among HIV-2 infected patients on ART, focusing on the immunological and virological responses in adults.

**Methods:**

Data were extracted from articles that were selected after screening of PubMed/MEDLINE up to November 2012 and abstracts of the 1996–2012 international conferences. Observational cohorts, clinical trials and program reports were eligible as long as they reported data on ART response (clinical, immunological or virological) among HIV-2 infected patients. The determinants investigated included patients’ demographic characteristics, CD4 cell count at baseline and ART received.

**Results:**

Seventeen reports (involving 976 HIV-2 only and 454 HIV1&2 dually reactive patients) were included in the final review, and the analysis presented in this report are related to HIV-2 infected patients only. There was no randomized controlled trial and only two cohorts had enrolled more than 100 HIV-2 only infected patients. The median CD4 count at ART initiation was 165 cells/mm^3^, [IQR; 137–201] and the median age at ART initiation was 44 years (IQR: 42–48 years). Ten studies included 103 patients treated with three nucleoside reverse transcriptase inhibitors (NRTI). Protease inhibitor (PI) based regimens were reported by 16 studies. Before 2009, the most frequent PIs used were Nelfinavir and Indinavir, whereas it was Lopinavir/ritonavir thereafter. The immunological response at month-12 was reported in six studies and the mean CD4 cell count increase was +118 cells/μL (min-max: 45–200 cells/μL).

**Conclusion:**

Overall, clinical and immuno-virologic outcomes in HIV-2 infected individuals treated with ART are suboptimal. There is a need of randomized controlled trials to improve the management and outcomes of people living with HIV-2 infection.

**Electronic supplementary material:**

The online version of this article (doi:10.1186/1471-2334-14-461) contains supplementary material, which is available to authorized users.

## Background

Although human immunodeficiency virus type 1 (HIV-1) infection is responsible for most of the global AIDS pandemic, HIV type 2 (HIV-2) is not infrequent in West Africa and is an additional and important cause of burden of disease with a limited spread to other regions of the world [1–3]. Overall in West Africa, between 10 and 20% of HIV infections include HIV-2 with a significant proportion of dually infected or reactive HIV-1 + 2 individuals [4, 5]. Interestingly, the prevalence of HIV-2 infections seems to be declining in West Africa, although the reasons remain unclear [1, 6–9]. Compared to HIV-1, HIV-2 infection is characterized by a longer clinical asymptomatic latency period [10], a slower T lymphocyte CD4 (CD4) depletion [11, 12] and a lower plasma viral load (VL) [13, 14]. Nevertheless, HIV-2 infection can lead to clinical AIDS [15, 16] and death [17–19] and such patients may clearly benefit from antiretroviral therapy (ART).

The 2013 World Health Organization (WHO) guidelines recommended the combined use of either three nucleoside reverse transcriptase inhibitors (NRTIs) or two NRTIs plus one protease inhibitor (PI) as the initial ART regimen for HIV-2 infection in a public health approach, [20]. These guidelines were based on observational studies with limited data. Their application could lead to the unavailability of effective second-line agents for HIV-2 infected patients in areas with limited access to ART, since phenotypic cross-resistance with PIs as well as NRTIs is a significant issue for HIV-2 [21–25].

No randomized clinical trial has assessed the efficacy of specific ART regimens in treatment-naïve HIV-2–infected patients [26, 27]. However, observational cohort studies in developed countries [15, 28, 29] have reported different and generally poorer treatment responses in HIV-2 patients compared to HIV-1 patients. Similar results were reported in a larger cohort collaboration in West Africa [30]. Additionally, data from few cohort studies conducted in resource-limited settings, such as Senegal [23–25], Gambia [18, 31–33], Cote-d’Ivoire [34] are focused generally on treatment outcomes or genotyping resistance mutation in HIV-2 infected patients.

Data comparing different ARV regimens among HIV-2 patients are even scarcer. Only one European cohort study has reported better immunological and virological responses to ritonavir-boosted PI-containing ART in antiretroviral-naïve HIV-2–infected patients compared to three NRTIs [15]. Overall, there has been minimal evidence-based recommendation regarding the best use of ART for HIV-2 infection [20, 33, 35–37]. We initiated this systematic review on ART response among HIV-2 and HIV-1/HIV-2 dually infected patients, to describe the different ART options that have been used and the different outcomes of these treatments.

## Methods

We conducted this systematic review according to the criteria set forth by the Preferred Reporting Items for Systematic Reviews and Meta-Analyses (PRISMA) group [38].

### Eligibility criteria

All studies, without design, place or language restrictions, were considered if they met the following four selection criteria: 1) data on clinical response (death or worsening of WHO stage), 2) data on immunological or virological response or both, sorted by ART regimen, 3) at least five patients receiving each drug regimen, 4) an available abstract, an article or an oral poster presentation. We included retrospective and prospective studies that reported responses to ART among HIV-2 and HIV-1/HIV-2 dually infected patients whatever the first-line regimen received. We excluded the case series with less than five patients. We also excluded studies that only reported data on genotypic analysis or only the prevalence of HIV-2 infection, or natural history.

### Search strategy and study selection

We developed a sensitive search strategy that combined terms for HIV-2 and ART (HAART or antiretroviral therapy or highly active or antiretroviral or therapy or highly active antiretroviral therapy, drug resistance, viral drug resistance).

*(“hiv-2”[MeSH Terms] OR “hiv-2”[All Fields] OR “hiv 2”[All Fields]) AND (“drug resistance, viral”[MeSH Terms] OR (“drug”[All Fields] AND “resistance”[All Fields] AND “viral”[All Fields]) OR “viral drug resistance”[All Fields] OR (“drug“[All Fields] AND “resistance“[All Fields] AND “viral“[All Fields]) OR “drug resistance, viral“[All Fields]) AND (“1996“[PDAT]: “2012“[PDAT]):*

*(“hiv-2“[MeSH Terms] OR “hiv-2“[All Fields] OR “hiv 2“[All Fields]) AND (“antiretroviral therapy, highly active”[MeSH Terms] OR (“antiretroviral”[All Fields] AND “therapy”[All Fields] AND “highly”[All Fields] AND “active”[All Fields]) OR “highly active antiretroviral therapy”[All Fields] OR “haart“[All Fields]) AND (“1996“[PDAT]: “2012“[PDAT])*

Initial searches were developed (DKE) for the following databases (from 1996 to November 1st, 2012): MEDLINE via PubMed, EMBASE, LILACS, Web of Science, Current Controlled Trials (http://www.controlled-trials.com), and the Cochrane Central Register of Controlled Trials. MEDLINE search was subsequently updated to November 1st, 2012. We also searched the data available on websites of International AIDS Society (IAS) conferences and of the Conference on Retroviruses and Opportunistic Infections (CROI) and International Conference for AIDS in Africa (ICASA). We particularly searched for abstracts from conferences held between July 2009 and July 2012 in order to identify studies that were recently completed but were possibly not yet published as full text articles. Bibliographies of relevant review articles and other papers were also screened. One of the authors (DKE) did a preliminary search, scanning all titles for eligibility according to the predefined inclusion criteria. The full abstracts of potentially eligible studies were then scanned by additional two reviewers (PC, JT) who worked independently to select potentially relevant full-text articles. Once all relevant full-text articles were reviewed, final agreement on study inclusion was determined through consensus (PC, DKE, JT and SPE).

### Data extraction and quality assessment

To decide whether or not the eligible studies met the inclusion criteria, each report was assessed by two independent reviewers (DKE, PC) using a standardized selection form developed for this purpose. Disagreements between observers were resolved by discussion. Data extraction was also conducted by the same reviewers using a standardized data extraction form created for this study and with the collaboration of external experts, when needed (JT, SPE). The following information was obtained from each study: first author’s name, journal and year of publication/presentation, design of the study, patient characteristic at baseline, location of the study, ART details, baseline median plasma HIV-2 VL, baseline CD4 count and length of follow-up. Each cohort was divided into categories according to ART used: PI-based regimen vs three NRTIs or other.

### Outcome measures

The outcomes of interest were the immunological response at 6, 12, 24 and 36 months (or equivalent time in weeks), the virological response at 12 and 24 months (as proportions at each time point) and the clinical progression including morbidity, ART discontinuation and mortality. The immunological and virological responses were considered when at least two measures of CD4 count or viral load at different moment were provided (one measure at baseline and at least one measure during follow up).

### Data analysis

A wide variation in definitions, outcomes, and specific components of ART response evaluated in the studies was observed. This did not allow us to aggregate statistical analysis of findings beyond a basic descriptive level. We therefore began by describing each study, identifying the ART regimens initiated. We also described the different outcomes reported in each study. Where possible, we used the reported data to compute a 95% confidence interval (CI) for mortality, immunological response (increase of CD4 cell count) and virological response (proportion of patients with VL below the detectable threshold). We were not able to summarize the viral resistance mutations into one main result by lack of standardization. We used STATA® software to estimate the median with the inter-quartile range (IQR) for the quantitative variables.

### Ethics statement

This systematic review was based on the data extraction of the articles published and was not therefore submitted to any ethic committee for a clearance.

## Results

### Study characteristics

**Figure 1:**
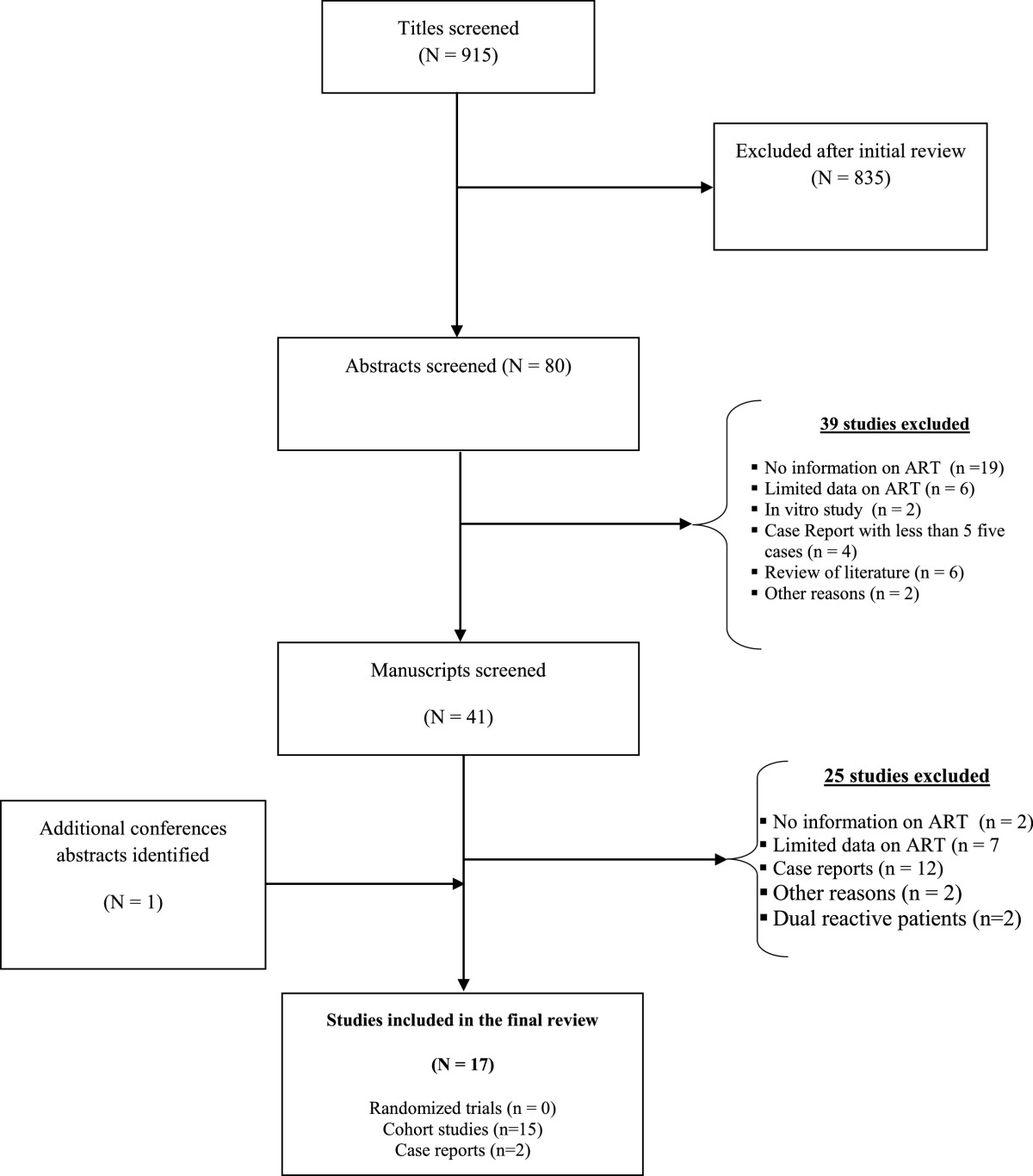
**Flow chart of the systematic review of antiretroviral therapy (ART) response in HIV-2 infected patients.**

Our search identified 915 papers and abstracts after removing duplicates. Of these, 835 were excluded on the basis of title and 39 others were excluded on the basis of content of the abstracts. Finally, after a full text screen, 25 reports were excluded because of insufficient information or target population only made up of HIV-1&2 dually reactive patients. One additional report from CROI web site was added. Altogether, 17 reports were included in the final review according to our eligibility criteria (Figure 1). There were no randomized trials, 15 cohort studies and two case series. Although the epicentre of HIV-2 is West Africa, it contributed only 8 studies, one was conducted in India, another in the USA, six in Europe and one study failed to adequately report the location. Ten studies involved HIV-2 infected patients only and the remaining included at least two or three sub-groups (HIV-2, HIV-1 and/or HIV-1&2 dually reactive) (Table 1).

**Table 1:** **Study characteristics of patients initiating antiretroviral therapy in 17 studies**

Study (Author, year)	Country	Sample size	Design	Population studied	Age* Median (IQR) years	% Male*	CD4 count at baseline*	Viral Load	ARV therapy* regimens
				(HIV-1, HIV-2, dual seropositive)				Median log 10 copies*	
Adje-Touré, 2003 [34]	Côte D’Ivoire	18	Cohort study	HIV-2 (n = 18)	41 [36–47]	78%	82 [52–188]	4.5 [4.1-5.2]	83% PI-based regimen (80% Nelfinavir)
Van Der Ende, 2003 [29]	The Netherlands	20	Cohort study	HIV-2 (n = 20)	50	62%	90 [10–360]	NR >5 log_10_ copies (38%)	80% on PI-based regimen
Mullins 2004 [39]	USA	10	Case series	HIV-2 (n = 9)	43 [35–44]	78%	134 [93–205]	NR	44% on PI-based regimen; 22% on PI-boosted regimen and 11% on NNRTIs
Matheron, 2006 [40]	France	61	Cohort study	HIV-2 (n = 61)	NR	NR	136 [57–244]	36 patients 3.1 [1.7-4.2]	77% PI-based regimen 23% 3NRTIs
Ndour, 2006 [41]	Senegal	188	Cohort study	HIV-2 (n = 35)	41 [29–61]	46%	204 [12–1029]	NR	100% on PI-based regimen (100% on Indinavir)
				HIV-1 (n = 153)					
Drylewicz, 2008 [12]	France	122	Cohort study	HIV-1 (n = 59)	NR	44%	267 [163–381]	34%, VL <2.7	58% on PI-based therapy 35% on LPV-r
				HIV-2 (n = 63)				2.9 [2.4-3.7]	
Ruelle, 2008 [42]	Belgium Luxembourg	22	Cohort study	HIV-2 (n = 20)	42	52%	226 [124–359]	4.1 [3.4-4.8]	68% on PI-based regimen 32% on 3 NRTIs-
Benard, 2009 [28]	France	29	Cohort study	HIV-2 (n = 29)	48 [43–55]	52%	142 [59–259]	3.3 [3.0-3.8]	96% PI-based regimen (100% on LPV-r) 4% 3 NRTIs
Jallow, 2009 [31]	Gambia	20	Cohort study	HIV-2 (n = 12) Dual (n = 8)	41 [31–47]	35%	145 [65–210]	4.9 [4.6-5.2]	100% PI-based regimen (100% on LPV/r)
Gottlieb, 2009 [23]	Senegal	23	Cohort study	HIV-2 (n = 23)	49 [31–60]	48%	200 [12–562]	30% VL <1.4 2.0 [<1.4-4.3]	96% on PI-based regimen (100% on Indinavir)
Harries, 2010 [17]	Burkina-Faso	4255	Cohort study	HIV-2 (n = 91) HIV-1 (n = 4043) Dual (n = 121)	44 [37–50]	39%	208 [103–459]	Not available	70% on PI-based regimen (27% LPV-r), 1% on 3NRTIS 29% on NNRTI-based regimen
Drylewicz, 2010 [30]	West Africa 5 countries	9482 (270 HIV-2)	Cohort study	HIV-2 (n = 270) HIV-1 (n = 9482) Dual (n = 321)	43 [36–50]	46%	148 [77–232]	Not available	71% PI-based regimen (31% on boosted PI)
Smith 2010 [43]	Senegal	74	Cohort study	HIV-2 (n = 74)	46 [21–64]	32%	202 [2–1124]	2.5 [1.6 – 4.3]	AZT 3TC IDV 62% AZT ATC LPV/r 12%; 3TC D4T IDV 9%; 3TC D4T LPV/Ir 5%; 3TC TDF LPV/Ir 3% 2–3 NRTI 2%; 2 NRTI 1 NNRTI 3%
Chiara, 2010 [44]	India	443	Cohort study	Dual (n = 4) HIV-2 (n = 25) HIV- (n = 414)	45 [41–49]	66%	95 [73–111]	NR	3 NRTIs (40%) PI-based regimen (60%)
**Study (Author, year)**	**Country**	**Sample size**	**Design**	**Population studied (HIV-1, HIV-2, dual seropositive)**	Age* Median (IQR) years	% Male*	CD4 count at baseline*	Viral Load (undetectable) Median log 10 copies*	Regimens ARV therapy*
Peterson, 2011 [18]	Gambia	352 (51 HIV-2)	Cohort study	HIV-1 (n = 308) HIV-2 (n = 51)	42 [32–48]	37%	140 [50–310]	4.9 [4.2-5.4]	88% PI-based regimen (100% LVP-r) 5% on NNRTI-based regimen 6% on 3 NRTIs
Benard, 2011 [15]	Europe 6 countries	170	Cohort study	HIV-2 (n = 170)	46 [39–52]	51%	N = 134 191 [90–275]	N = 110 39% VL <2.7 4.0 [3.4-4.6]	74% PI-based (61% on LPV-r) 26% 3NRTIs
Peterson, 2012 [45]	NR	5	Case series	HIV-2 (n = 5)	50 [41–55]	20%	181 [96–200]	NR	5 patients on raltegravir

*For HIV-2 infected patients only, NR: not reported, NA: not available.

Table 1 describes the characteristics of these 17 studies. Only two cohorts had enrolled more than 100 HIV-2 infected patients: one from Europe that recruited in six countries and enrolled 170 HIV-2 infected patients [15], and the other one from West Africa with the participation of sites in five countries and the enrolment of 270 HIV-2 infected patients [30]. For the remaining 15 studies, the sample size ranged from 5 to 91 HIV-2 infected patients.

The 17 reports contributed 1 430 patients, but only 976 were infected with HIV-2 only and constitute the core sample for this report. Their median age was 44 years (IQR: 42–48 years). All the studies selected provided a CD4 cell count at baseline except one from the Netherlands and the median CD4 cell count at ART initiation was 165 cells/mm^3^ (IQR: 138–203). Only six cohort studies reported a median CD4 cell count ≥200 cells/mm^3^ at baseline. The HIV-2 VL at baseline was reported in 10 studies (59%) and among them the median HIV-2 VL at ART initiation was 3.7 log_10_ copies/ml, IQR [2.9 - 4.5].

Table 2 describes the antiretroviral drug combinations used for the HIV-2 infected patients. Ten studies reported using three NRTIs for a total of 102 patients. Before 2009, the most frequently used PIs were Nelfinavir or Indinavir and Lopinavir/ritonavir-based regimens were used thereafter as heat-stable FDC tablets became widely available in West Africa..

**Table 2:** **First-line antiretroviral treatment initiated in HIV-2 infected patients in 17 studies**

Study	Study period	Dual NRTI	3 NRTIs	PI-based regimen				Boosted PI
(Author, year)		Therapy						
				LPV	NFV	IDV	SQV	
Adje-Touré, 2003 [34]	1998-2000	Yes (n = 6)	Yes (n = 1)	No	Yes (n = 7)	Yes (n = 4)	No	
Van Der Ende, 2003 [29]	1995-2001	Yes (n = 0)	Yes (n = 2)	No	Yes (n = 1)	Yes (n = 14)	Yes (n = 3)	80% boosted PI
Mullins 2004^$^[39]	1994-2003	No	Yes (n = 2)	No	Yes (2 )	Yes (n = 2)	Yes [3]	Ritonavir boosted PI (n = 2; 22%)
Matheron, 2006 [40]	NR- 2004	No	Yes (n = 14)	No	Yes (n = 17)	No	No	Ritonavir boosted PI (n = 23)
Ndour, 2006 [41]	1998-2004	No	No	No	No	Yes (n = 35)	No	No
Drylewicz, 2008 [12]	1996-2006	No	NR	Yes (n = 14)	Yes (n = 10)	Yes (n = 1)	No	Ritonavir-boosted PI (n = 24)
Ruelle 2008 [42]	NR-2006	No	Yes (n = 6)	Yes (n = 4)	Yes (n = 3)	Yes (n = 5)	Yes (n = 1)	4 NRTIs (n = 1) Ritonavir-boosted PI (n = 10)
Benard A, 2009 [28]	NR	No	No	Yes (n = 29)	No	No	No	Ritonavir-boosted (n = 29; 100%)
Jallow, 2009 [31]	2004-2009	No	No	Yes (n = 20)	No	No	No	Ritonavir-boosted (n = 20; 100%)
Gottlieb, 2009 [23]	2005-NR	No	No	No	No	Yes (n = 22)	No	No
Harries 2010 [17]	2002-2008	No	Yes (n = 1)	Yes (n = 17)	Yes (n = 35)	Yes (n = 12)	No	
Drylewicz 2010 [30]	1997-2007	No	Yes (n = 21)	NR	NR	NR	NR	PI = 193 Ritonavir-boosted (n = 84; 43%)
Smith 2010 [43]	NR	No	Yes (n = 1)	Yes (n = 0)	No	Yes (n = 53)	No	Ritonavir-boosted (n = 15; 22.4%)
Chiara, 2010 [44]	2006-2009	No	Yes (n = 10)	NR	NR	Yes (n = 15)	NR	Ritonavir-boosted (n = 15; 100%)
Peterson 2011 [18]	2004-2009	NR	NR	Yes [46]	No	No	No	Ritonavir-boosted PI (n = 45, 100%)
Benard, 2011 [15]	1998-2008	No	Yes (n = 44)	Yes (n = 76)	No	Yes (n = 18)	Yes (n = 16)	Ritonavir-boosted PI (n = 126)
Peterson, 2012 [45]	NR	Yes (n = 0)	No	Yes (n = 1)	Yes (n = 2)	Yes (n = 1)	Yes (n = 0)	One patient on raltegravir as first-line regimen

NR: not reported, NA: not available $ Only the last treatment received was considered

### Study outcomes

In our review, 14 studies (82%) reported immunological response, eight (47%) reported virological response, eight studies (47%) reported clinical events, and six (35%) reported data on loss to follow-up.

- *Mortality and loss to follow-up*
  A crude mortality rate was reported in eight cohorts without stratification by drug regimen (Table 3). The pooled crude mortality rate was 4.8% (37/771). Benard et al. in a French cohort study reported two deaths (6.9%) out of 29 HIV-2 infected patients followed up in median 26 months and treated with Lopinavir-ritonavir: one from a bladder cancer and another one from a lung cancer [28]. In the European cohort, no death was reported during the first 12 months of follow-up among 170 HIV-2 infected patients (126 initiated a PI-based regimen and 44 started with three NRTIs [15]). Studies from three developing countries reported mortality. Peterson in the Gambia reported six deaths among 51 HIV-2 infected patients (11.8%) followed up in median 20 months. In this latter study, the survival rate was 96% at 12 months and 80% at 36 months [18]. Smith in Senegal reported seven deaths among 74 HIV-2 infected patients (9.5%) followed up in median 13 months [43] and in Burkina Faso, Harries reported 14 deaths among 91 HIV-2 infected patients (15.4%) followed up in median 23 months [17]. Loss to follow-up was reported in six studies and varied between 0% in India [44] to 7.5% in Burkina-faso [17].

**Table 3:** **Death and AIDS Progression among HIV-2 –infected patients in 17 studies**

Study	Duration on follow up on ART (months)	Crude mortality	Survival	Survival	Survival	Progression to AIDS
(Author, year)			Month 12	Month 24	Month 36	
						(%)
Adje-Touré, 2003 [34]	11 [7–12]	NR	NR	NR	NR	NR
Van Der Ende, 2003 [29]	23 [13–58]	NR	NR	NR	NR	2/13 (15%)
Mullins 2004 [39]	22 [8 – 35]	NR	100%	NR	NR	NR
Matheron, 2006 [40]	21	5/61 (15.8%)	NR	NR	NR	3/61 (4.9%)
Ndour, 2006 [41]	10 [1–21]	NR	NR	NR	NR	NR* (opportunistic infections 9/35)
Drylewicz, 2008 [12]	NR	NR	NR	NR	NR	NR
Ruelle 2008 [42]	NR	NR	NR	NR	NR	NR
Benard A, 2009 [28]	26 [10–33]	2/29 (6.9%)	NR	NR	NR	NR
Jallow, 2009 [31]	NR	NR	NR	NR	NR	NR
Gottlieb, 2009 [23]	17 [4–55]	NR	NR	NR	NR	NR
Harries, 2010 [17]	23 [8–34]	14/91 (15.4%)	NR	NR	NR	NR
Drylewicz 2010 [30]	11 [6–13]	3/270 (1%)	NR	NR	NR	NR
Smith 2010 [43]	13 [0–40]	7/74 (9.5%)	NR	NR	NR	24 HIV-AIDS related events
Chiara, 2010 [44]	36	0/25 (0.0)	NR	NR	NR	NR
Peterson 2011 [18]	20 [10–33]	6/51 (11.8%)	96%	89%	80%	NR
			(89–100)	(76–100)	(58–100)	
Benard, 2011 [15]	20 [8–36]	0/170 (0%)	NR	NR	NR	Yes (n = 10)
		During the first 12 months				1/44 (2%) 3 NRTIs
						9/126 (7%): PI regimen
Peterson K, 2012 [45]	NR	NR	NR	NR	NR	NR

*Included only the studies which reported virological responses.

NR: Not Reported, VL: viral load.
- *AIDS progression*
  In the French cohort, none of the 18 HIV-2 patients with CDC stage A at baseline and treated with a PI-based regimen progressed to AIDS during follow-up. In the European Cohort, among the 170 HIV-2 infected patients enrolled (44 on three NRTIs), one patient (2%) receiving a triple NRTI regimen experienced progression to AIDS (tuberculosis) five months after treatment initiation. Among patients treated with PI/r, nine (7%) progressed to AIDS (cytomegalovirus infections [2], recurrent bacterial pneumonia [1], candidiasis [1], toxoplasmosis [1], cryptococcosis [1], pneumocystosis [1], HIV wasting syndrome [1], and unknown [1]) within a median delay of two months (min-max: 0.5–7.5 months) after treatment initiation [15].
- *CD4 response*
  The CD4 response was reported in 14 studies (Table 4). The median CD4 cell count increased at month-6 after ART initiation was +72 cells/μL (min-max: +41-140) cells/μL) based on four studies. In the French cohort [40], the median CD4 cell count did not differ between patients treated with a PI-containing regimen and those with three NRTIs at month-6 (P = 0.47) after treatment initiation. Always at month-6, in the group of patients without PI (n = 10), the median CD4 cell count increased was +57 (min-max: +37; +100) cells/μL whereas it was +52 (min-max: +8; +81) cells/μL among the 40 HIV-2 infected patients who had initiated a PI-containing regimen.

**Table 4:** Immunological responses* among HIV-2 –infected patients in 17 studies

Study (Author, year)	CD4 count Baseline Median [IQR]	CD4 count Month-6 Median [IQR]	CD4 count Month-12 Median [IQR]	CD4 count Month -24 Median [IQR]	Delta of CD4 count cells/mm^3^	Slope of CD4 count (first period)	Slope of CD4 count (second period)	Comments
Adje-Touré, 2003 [34]	82 [52–188]	154 [68–275]	163 [132–244]	NR	NR	NR	NR	NR
Van Der Ende, 2003 [29]	90 [10–360]	230 [40–380]	270 [60–410]	NR	NR	NR	NR	13 patients (Group II)
Mullins 2004 [39]	134[93–205]	NR	NR	NR	NR	NR	NR	Median of CD4 count at the last visit
								327 [202–408]
Matheron, 2006 [40]	136 [57–244]	177	181	221	M6: +53 (+10; +86)	NR	NR	*At 12 months*
		[98–328]	[123–290]	[133–374]				*No PI*: +40 (+18; +97)
					M12: +41 (+9; +92)			
					M24: +62 (9; 120)			*PI*: (+41 (+8, +86)
								P (0.67)
Ndour, 2006 [41]	204 [12–1029]	NR	NR	NR	Month-12	NR	NR	NR
					+200 cells/mm^3^			
Drylewicz, 2008 [12]	267 [163–381]	NR	NR	NR	NR	<2 months 25 (7; 42) cells/μl/months	>2 months-3 (-38; +32) cells/μl/year	Slope of % of CD4 count was reported
Ruelle 2008 [42]	226 [124–359]	NR	NR	NR	*PI containing regimens*: +89 cells/mm^3^ [-31; 323]		PI group 106 cells/μl/year	
					*PI sparing regimens*-53 cells/mm^3^ [-62; 57]		Without PI-25 cells/μl/year	
Benard A, 2009 [28]	142 [59–259]				M6: +71 [11–116] M12: +122 [61–159] M24 + 132 [110–275]	<3 months 24.3 (7.1; 41.4) cells/μl/months	>3 months +8.50 (+5.3; +11.7) cells/μl/year	
Jallow, 2009 [31]	145 [65–210]	NR	NR	NR	NR	NR	NR	NR
Gottlieb, 2009 [23]	200 [12–562]	NR	NR	NR	NR	NR	NR	NR
Harries, 2010 [17]	111 [31–171]	NR	NR	NR	NR	NR	NR	Means CD4 count M6 = 255, M12 = 270
Drylewicz 2010 [30]	178 [77–232]	NR	PI 278 [248–307] NNRTIs 268 [175–293]	NR	NR	<3 months NNRTIs: -41 (-123; 40) cells/μl/year	>3 months +8.50 (+5.3; +11.7) cells/μl/year	
Smith 2010 [43]	202 [2–1124]	NR	NR	NR			+8 [-100; +181] cells/mm^3^/year	64% CD4 increase and 37% cd4 decline in follow-up
Chiara, 2010 [44]	96 [73–111] (3NRTIs)/PI (114)/78	3NRTIs/PI (102)/171	3NRTIs/PI (91)/254	NR	NR	NR	NR	NR
Peterson 2011 [18]	140 [50–310]	NR	NR	NR	M6: +120 cells M12: +115 cells M24: +285 cells M36: +280	NR	NR	NR
Benard, 2011 [15]	216 [150–287] *PI:* 191 [90–275] *3NRTI:* 170 [72–275]	NR	*PI (*327) *3NRTI (*191)	NR	NR	*<3 months* PI: +12 cells/μl/months 3NRTI: +6 cells/μl/months	*3-12 months* PI: +76 cells/μl/y 3NRTI: -60 cells/μl/y	> = 200 cells/mm^3^ PI: +52 cells/μl/y 3NRTI: -99 cells/μl/y
Peterson K, 2012 [45]	181 [96–200]	NR	NR	NR	NR	NR	NR	NR

*Included only the study who reported immunological responses.

NR: Not Reported.
- Overall, the median CD4 cell count increase at month 12 after ART initiation was +118 cells/μL (min-max: +45-200) cells/μL based on six studies. In France, at month 12 in the group of patients without PI (n = 9), the median CD4 cell count increase was +71 (min-max: +0; +90) cells/μL whereas it was +58 (min-max +11; +130) cells/μL among the 29 patients who had initiated a PI-containing regimen [40].
- Only one study reported the immunological response at month-12 per drug regimen [15]. After three months of treatment, the estimated CD4 cell count decreased in patients treated with three NRTIs and increased in those treated with PI/r (-60 vs 176 cells/mm^3^/year in median; p = 0.002). These changes resulted in estimated CD4 cell counts at month 12 being lower in patients treated with three NRTIs than in patients treated with PI/r (191 vs 327 cells/mm^3^ in median; p = .001). The difference in estimated median CD4 cell counts at month 12 between patients treated with three NRTIs and those treated with a boosted PI-containing regimen remained statistically significant after adjustment for geographical origin (p = 0.0009) or for baseline HIV-2 RNA level (p = 0.05) [15].
- *Virological response*
  The virological response was reported in 11 studies (Table 5). The threshold of detection of HIV-2 VL varied from one study to another (min-max: 1.4-2.7 log_10_) (Table 5). Overall, among HIV-2 infected patients who initiated ART and had VL data available, 10% to 39% had an undetectable VL at baseline. In the Gambia, 81% of patients enrolled had their VL <400 copies/mL [18]. Ruelle et al. [42] in Belgium and Luxembourg reported that eight out of 13 HIV-2 infected patients (62%) had undetectable VL among patients who initiated a PI-based regimen. On the other hand, among patients who initiated a regimen without PI, one out of six (17%) had an undetectable VL at baseline. Three studies reported virological response data in patients who initiated a PI-based regimen or another regimen. Matheron et al. [40] reported a median change of VL of -1.0 (IQR -1.0; 00) log_10_ copies/ml among patients without PI whereas it was -0.6 (-1.7, 00) log_10_ copies/ml among patients who initiated a PI-based regimen. A similar report in the European cohort indicated a change of -1.8 log_10_ copies/ml among patients initiating a PI-based regimen and 0 log_10_ among patients who initiated ART with three NRTIs [15]. The proportion of patients with undetectable VL at 36 months was 81.4% in the Gambian study [18].

**Table 5:** Virological responses* among HIV-2 –infected patients in 17 studies

Study	VL	VL	VL	VL	Delta of VL	Slope of VL (first period)	Slope of VL (second period)	Other response
(Author, year)	Threshold	Baseline	Month-12	Month -24				
	% non detectable	N, Median [IQR]	n, Median [IQR]	n, Median [IQR]				
Adje-Touré, 2003 [35]	NR	4.5 (4.1-5.2)	4.1 (3.2-4.9)	NR	NR	NR	NR	
Van Der Ende, 2003 [28]	<2.7 log_10_ (10%)	NR	NR	NR	NR	NR	NR	At week 23, 13/18 (72%) had VL <2.7 log_10_
Mullins 2004	<2.0 cp log_10_	NR	NR	NR	NR	NR	NR	Median viral load at the last visit 3.51 [3.27-4.43] log_10_
Matheron, 2006 [47]	NR	3.1 (1.7-4.2)	1.7 (1.7-2.9)	1.7 (1.7-1.7)	*12 months:* -0.6 (-1.7; 0.0)			
					*24 months*			
					-0.6 (-2.5; 0)			
Ndour, 2006 [41]	NR	NR	NR	NR	NR	NR	NR	NR
Drylewicz, 2008 [12]	<2.7	2.9	NR	NR	NR	*<2 months*	*>2 months*	
	(34%)	(2.4-3.7)				-0.62 (-0.84; - 0.40) log_10_ cp/ml/month	0.02 (-0.27; 0.32)	
							log_10_ cp/ml/year	
Ruelle 2008 [46]		4.1	PI based regimen 8/13 (62%) undectable Without PI 1/6 (17%) undectable	PI based regimen 5/8 (63%) undectable Without PI 1/4 (25%) undectable				
		(3.4-4.8)						
Benard A, 2009 [26]	NR	3.3			*3 months* (n = 20) 80% had undectable VL			
	N = 24 (33%)	[3.0-3.8]						
Jallow, 2009 [32]	<2.0 log_10_	4.9 [4.6-5.2]	NR	NR	NR	NR	NR	NR
Gottlieb, 2009 [29]	<1.4 log_10_ N = 23 (30%)	2.0 [<1.4-4.3]	NR	NR	NR	NR	NR	*No resistance*
								9/11 (82%) had VL <1.4
								*Resistance* 3/12 (25%) had VL <1.4
Harries, 2010 [17]	NR	NR	NR	NR	NR	NR	NR	NR
Drylewicz 2010 [36]	NR	NR	NR	NR	NR	NR	NR	NR
Smith 2010 [42]	<1.4 log_10_	2.5 [1.6-4.3]	NR	NR	NR	NR	NR	35% (n = 74) had detectable RNA plasma HIV-2 at their last follow-up visit. Median 100 [31–1997] copies/ml
Chiara, 2010 [48]	NR	NR	NR	NR	NR	NR	NR	NR
Peterson 2011 [18]	NR	4.9 [4.2-5.4]	81% VL <400	89% VL <400	NR	NR	NR	19% expressed viral rebound by 36 months
Benard, 2011 [15]	<2.7 log_10_	PI: 4.0 [3.4-4.6] 3NRTIs 4.0 [2.9-4.6]	PI: 2.2 3NRTIs 4.0	NR	NR	*0-3 months*	*3-12 months*	
	N = 110 (39%)					PI: -0.3 log_10_/ml/months	PI: -0.002 log_10_/ml/months	
						3 NRTIs: -0.2 log_10_/ml/months	3 NRTIs: - +1.6 log_10_/ml/months	
Peterson K, 2012 (56)	NR	NR	NR	NR	NR	NR	NR	NR

*Included only the studies which reported virological responses.

NR: Not Reported, VL: viral load.

## Discussion

This systematic review illustrates the heterogeneity of the reports of treatment outcomes of HIV-2 infected patients initiating ART, especially in resource-limited settings. Therefore, the global response on ART among HIV-2 infected patients remains difficult to synthesize. By the end of 2012, 17 publications reported usable information and only one study reported outcomes stratified by baseline CD4 cells count [15]. Our study highlights the need for standardized reporting of ART outcomes among HIV-2 infected patients akin those living with HIV-1.

To date, the use of VL for ART monitoring and initiation in HIV-2 infected patients has been a challenge for two reasons. First, there is no US or European-approved plasma VL test for HIV-2 infection, although assays are becoming increasingly available [47, 48], second, many HIV-2 infected patients eligible for ART have an undetectable VL. For example, in the European cohort, 39% of HIV-2 infected patients had an undetectable VL, though the median CD4 cell count at baseline was 191 cells/mm^3^ [IQR: 90–275]. Moreover, based on our recent experience in West Africa among patients with CD4 counts <500 cells/mm^3^, 76% and 47% of patients had an undetectable VL when considering thresholds of 50 copies/ml and 10 copies/ml, respectively [47]. Hence, it is more appropriate at this stage to use patients’ CD4 cell count and clinical stage for decision on ART initiation, as it has originally been the standard for HIV-1. However, there is no consensus on the level of CD4 cells count at which to start treatment for HIV-2. The US [37] and British ART guidelines [35] do not provide specific recommendations on the level of CD4 cells count at which ART should be initiated among asymptomatic HIV-2 infected patients. The 2010 French guidelines were the first ones to recommend ART initiation when the CD4 cell count was below 500 cells/mm^3^[46]. The WHO recommended the same threshold for HIV-2 and HIV-1 infected patients (CD4 < 350cells/ mm^3^) in the 2010 version [49] and now the same threshold for HIV-2 and HIV-1 (CD4 < 500 cells/mm^3^) in the recent 2013 recommendation as there was no evidence showing the benefit to start earlier in HIV-2 infected patients [20].

However in most of the reports reviewed, there seems to be a poorer CD4 cell count recovery after treatment initiation in HIV-2 infected patients compared to the HIV-1 infected ones [12]. This systematic review reveals that the median CD4 cell count at ART initiation was 165 cells/mm^3^ (IQR; 137–202), which is very close to the median CD4 count among HIV-1 infected patients in the same settings [12, 30]. Furthermore, the median age at ART initiation in this report was 44 years for HIV-2 infected patients (IQR; 42–46 years) and 37 years for the HIV-1 infected ones (Table 1) [30]. In older HIV-1-infected patient on ART, a poorer immunological response has previously been reported compared to younger ones in the same part of the world [50]. It can thus be assumed that poor immunological responses could also be expected among older HIV-2 infected patients. All the aforementioned argue in favor of early ART initiation among HIV-2 infected patients.

PI-based regimens (usually LPV/r) remains the first-line therapy most prescribed among HIV-2 infected patients in accordance with the different guidelines available [33, 35–37]. Data on three NRTI-based regimens, an alternative for lower-income countries in the context of the high prevalence of tuberculosis, are limited. The lack of large observational or randomized treatment studies in HIV-2 infected patients makes it difficult to decide at this stage when and which therapy should be started [26, 27]. Hence, there is an urgent need for randomized controlled trials to define the best sequencing of ART among HIV-2 infected patients, specifically in areas with limited access to second-line therapy based on alternative HIV-2 active PIs (Darunavir/r or Saquinavir/r) or integrase inhibitor-based regimens. In addition, optimizing the NRTI backbone in patients failing first-line regimens is an area that needs to be explored as data suggest a low barrier to class-wide NRTI resistance [51]. There is also no report on the switch of first-line regimens among HIV-2 infected patients. This could be explained by a lack of clear definition of treatment failure among HIV-2 infected patients [20, 35–37]. In HIV-2 infected patients with virological failure of first-line or subsequent regimens, genotypic resistance testing may be beneficial but interpretation algorithms are not well validated for most ARVs [52]. With this respect, introduction of new drugs and drug classes in countries with limited resources should be seriously considered.

This is to our knowledge the first systematic review on ART responses among HIV-2 infected patients including data from Europe, India and Africa. This review provides an overview of the different therapeutic strategies that have been used for HIV-2 infected patients so far, and their main outcomes, often documented in resource-limited settings but with limited evidence-based conclusions. Nonetheless, our review of available data should help to guide future studies and preferably clinical trials among HIV-2 infected patients.

We found substantial variations of ART responses reported over time and we were thus unable to identify any preferred ART regimen for HIV-2 infected patients. Only one study compared two different treatment regimens (PI-based vs. three NRTIs) [15]. In addition, the substantial heterogeneity in results observed between studies made it more difficult to determine the magnitude of the relative influence of individuals’ characteristics on treatment response. The main limitation of this review is the use of several patient populations probably overlapping each other, such as ANRS studies [12, 28, 53] and the Senegalese studies [25, 39] and the two international collaborative studies ACHIEV_2_E in Europe [15] and IeDEA West Africa [30, 54] Moreover, it was also challenging to analyze data in this review because each study reported the main outcomes in different manner. For example the CD4 count response was presented as an absolute difference delta or as a slope of CD4 count at different time points. It is therefore advisable to harmonize data presentation for future reports on ART response among HIV-2 infected patients. For example, CD4 slopes should be systematically reported as performed by the European and West Africa Collaborations [12, 15, 30]. For all the above, we were unable to pool the data extracted with different outcomes reported at different times and the overlapped study populations; hence we could not perform a meta-analysis.

## Conclusion

In summary, our review did not find clear evidence on the response to different ART regimens used for HIV-2 infection mainly based on CD4 counts. This observation provides further justification to conduct randomized controlled trials among HIV-2 infected patients.

## Authors’ original submitted files for images

Below are the links to the authors’ original submitted files for images. Authors’ original file for figure 1
