# Supplementary figures and images for: Antiretroviral therapy response among HIV-2 infected patients: a systematic review
Source: BMC Infect Dis. 2014 Aug 26;14:461. doi: 10.1186/1471-2334-14-461 (PMC4156654; doi:10.1186/1471-2334-14-461)

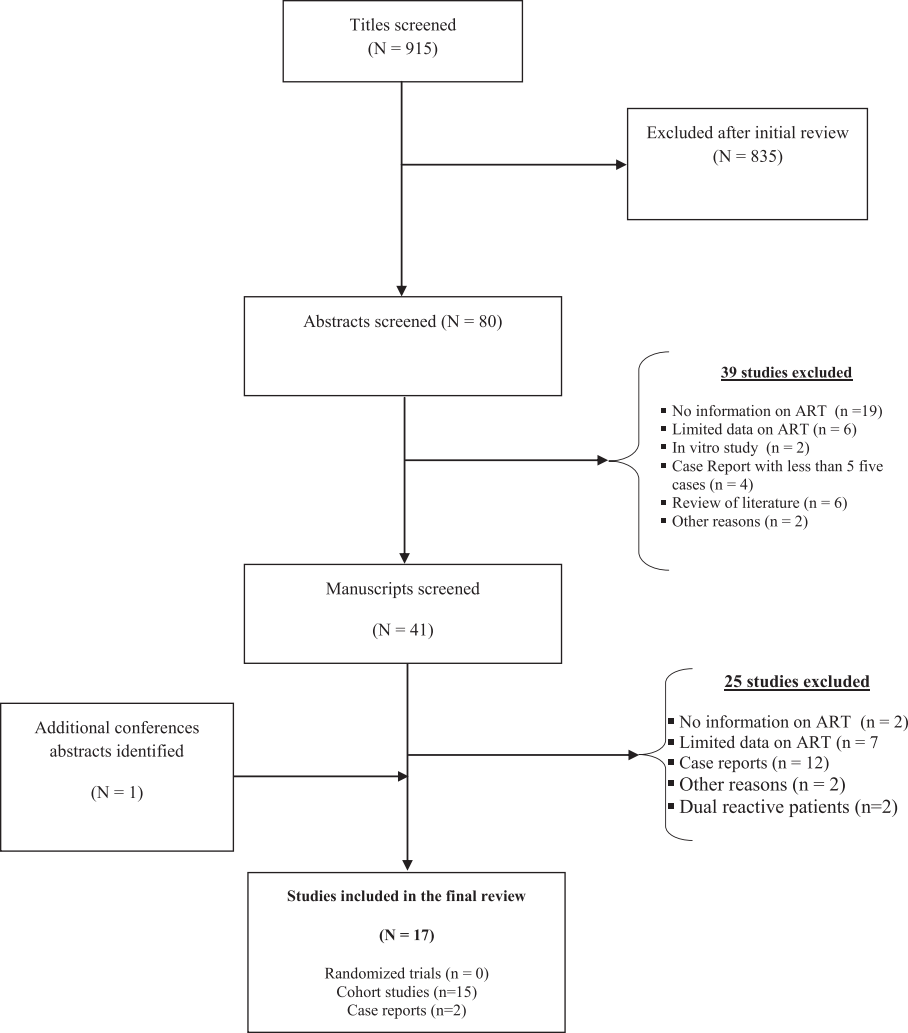

Supplement: Supplementary file 1 — Authors’ original file for figure 1 [file 12879_2013_3764_MOESM1_ESM.pdf]
